# Combination of Chemotherapy and Mild Hyperthermia Using Targeted Nanoparticles: A Potential Treatment Modality for Breast Cancer

**DOI:** 10.3390/pharmaceutics15051389

**Published:** 2023-04-30

**Authors:** Ishdeep Kaur, Terence Tieu, Veerasikku G. Deepagan, Muhammad A. Ali, Fahad Alsunaydih, David Rudd, Maliheh A. Moghaddam, Laure Bourgeois, Timothy E. Adams, Kristofer J. Thurecht, Mehmet Yuce, Anna Cifuentes-Rius, Nicolas H. Voelcker

**Affiliations:** 1 Monash Institute of Pharmacy and Pharmaceutical Sciences, Monash University, 381, Royal Parade, Parkville, VIC 3052, Australia; i.ishdeepkaur@gmail.com (I.K.);; 2 Department of Electrical and Computer Systems Engineering, Monash University, Clayton Campus, Clayton, VIC 3168, Australia; 3 Centre of Polymer Systems, Tomas Bata University, 5678, 760 01 Zlin, Czech Republic; 4 Monash Centre for Electron Microscopy, Clayton Campus, Monash University, Clayton, VIC 3168, Australia; 5 Commonwealth Scientific and Industrial Research Organization (CSIRO), 343, Royal Parade, Parkville, VIC 3052, Australia; 6 Australian Institute for Bioengineering and Nanotechnology (AIBN), Corner College and Cooper Rds, The University of Queensland, Brisbane, QLD 4072, Australia; 7 Melbourne Centre for Nanofabrication, Victorian Node of the Australian National Fabrication Facility, Clayton, VIC 3168, Australia

**Keywords:** porous silicon nanoparticles, drug delivery, hyperthermia, breast cancer, combination therapy

## Abstract

Despite the clinical benefits that chemotherapeutics has had on the treatment of breast cancer, drug resistance remains one of the main obstacles to curative cancer therapy. Nanomedicines allow therapeutics to be more targeted and effective, resulting in enhanced treatment success, reduced side effects, and the possibility of minimising drug resistance by the co-delivery of therapeutic agents. Porous silicon nanoparticles (pSiNPs) have been established as efficient vectors for drug delivery. Their high surface area makes them an ideal carrier for the administration of multiple therapeutics, providing the means to apply multiple attacks to the tumour. Moreover, immobilising targeting ligands on the pSiNP surface helps direct them selectively to cancer cells, thereby reducing harm to normal tissues. Here, we engineered breast cancer-targeted pSiNPs co-loaded with an anticancer drug and gold nanoclusters (AuNCs). AuNCs have the capacity to induce hyperthermia when exposed to a radiofrequency field. Using monolayer and 3D cell cultures, we demonstrate that the cell-killing efficacy of combined hyperthermia and chemotherapy via targeted pSiNPs is 1.5-fold higher than applying monotherapy and 3.5-fold higher compared to using a nontargeted system with combined therapeutics. The results not only demonstrate targeted pSiNPs as a successful nanocarrier for combination therapy but also confirm it as a versatile platform with the potential to be used for personalised medicine.

## 1. Introduction

Breast cancer (BCa) is one of the leading causes of death among women. Chemotherapy is a common treatment approach to treat BCa besides surgery, radiation, hormone, and targeted therapy [1]. Chemotherapeutic drugs have improved patient outcomes but are associated with various side effects due to systemic toxicity and susceptibility to chemoresistance development over time and duration of the treatment, allowing tumours to reinitiate growth and spread, with devastating consequences for patients [2,3].

Nanomedicines have improved the delivery of chemotherapeutics, resulting in an enhanced therapeutic effect [4]. By using nanomaterials as drug delivery vectors, increased administration, and availability (especially for insoluble drugs) [5], drug accumulation to the tumour site [6], and nontoxic side effects [7] have been achieved. Porous silicon nanoparticles (pSiNPs)—inorganic nanocarriers with well-defined pores—offer biodegradable and biocompatible properties, high loading capacity of multiple therapeutics given due to the large internal surface area of the nanocarrier, and tuneable sustained release kinetics, which are key requirements in nanomedicine design [8].

Furthermore, pSiNPs can be chemically modified with targeting moieties such as antibodies, peptides, and aptamers to induce active targeting of cancer cells [9]. The addition of these active targeting moieties onto the outer nanoparticle surface can further enhance their accumulation and internalisation into the tumour and target cells. Moreover, targeted therapies can help mitigate toxicity to normal cells by targeting specific genes, proteins or the tissue environment that contribute to cancer growth and survival [10,11,12,13]. For example, trastuzumab, a monoclonal antibody against nonmetastatic human epidermal growth factor receptor 2 (HER2) BCa, was the first approved generic drug targeting HER2 receptors to stop cancer cell growth and division [14]. Unfortunately, there are not many options available for other BCa subtypes, such as triple negative BCa (TNBC) and metastatic forms of cancer. TNBC lacks the three most common receptors found in BCa, estrogen, progesterone, and HER2 receptors. To overcome this, we have recently shown that TNBC primary tumour growth and, particularly, the metastatic spread, can be reduced in a bioengineered mouse model using drug-loaded pSiNPs targeting epidermal growth factor receptor (EGFR)—overexpressed in this BCa subtype—demonstrating the potential of pSiNPs as a drug delivery vector [15].

While active targeting has the potential to reduce toxicity to healthy cells and organs, multidrug resistance is still a major challenge to overcome, diminishing a long-term therapeutic effect [16].To avoid drug resistance, clinicians have started to treat patients using multiple drugs [17,18]. However, administering multiple therapeutics can add to combinatorial toxicity issues and pharmacokinetic differences that are hard to predict [19,20]. Therefore, developing versatile drug delivery platforms that can (a) actively target overexpressed receptors on the cancer cell surface and (b) deliver multiple therapeutics in combination represents a promising strategy to overcome the hurdles of current chemotherapeutics.

Applying a combination of therapies through the same nanoparticle-based delivery system has several advantages, such as reduced drug-related toxicity due to the requirement of a lower drug concentration and overcoming drug resistance by modulating different signalling pathways at once [21,22].

Combining hyperthermia and chemotherapy is one such example and has shown to be beneficial for cancer therapy [23,24]. Clinically, hyperthermia involves exposure to elevated temperatures in the range of 39–45 °C [25]. Heating at milder temperatures (39 °C–42 °C) enhances the blood flow of the tumour site and can therefore facilitate the access of nanoparticles and drugs to the otherwise hard-to-penetrate tumour microenvironment [26]. Temperatures above 42 °C result in time and temperature-dependent cell toxicity [27] The generation of hyperthermia causes several cellular changes, such as protein denaturation and aggregation, and the loss of cellular homeostasis, resulting in cell cycle arrest [25]. Additionally, at such temperatures, a generation of reactive oxygen species (ROS) can cause chromosomal damage and inhibit DNA repair hindering various cellular processes and inducing cell death—which can be exploited as a chemosensitiser [25]. Besides chemotherapy, several combination therapies with hyperthermia have been explored for breast cancer treatment previously, including the use of photodynamic therapy [28,29] and immunotherapy [30,31]. However, we explored the application of hyperthermia in combination with chemotherapy as it offers the significant advantage of requiring lower doses of drugs, leading to an effective treatment with reduced side effects and resistance to drugs by cancer cells [32].

A noninvasive approach to producing hyperthermia consists of electromagnetic radiation in the presence of metallic nanoclusters, which results in heat generation [33]. Utilising radiofrequency (RF) waves with high penetration depth as a harmless and nonionising energy band within the electromagnetic spectrum is advantageous in developing a hyperthermia system [34]. Gold nanoclusters (AuNCs) of smaller than 3 nanometres in size have attracted interest due to their unique properties [35]. The quantum confinement effect facilitates the discreet electronic structure of the nanoclusters, which results in a series of molecular-like properties such as fluorescence and paramagnetism [36,37]. We have shown that AuNCs interact with RF fields in the microwave region (at 1 GHz) generating hyperthermia-mediated cell death in lymphocyte cells [38], which results in an excellent chemosensitiser [33]. This is because AuNCs can produce heat under an inductively coupled RF apparatus at microwave frequencies [39]. Furthermore, compared to larger gold nanoparticles, AuNCs have been reported to have longer blood circulation times [40] and higher penetration depth in tumour tissue—both advantageous in the development of nano-enabled anticancer therapies [41,42].

In this study, we fabricated multifunctional pSiNPs loaded with both anticancer drugs and AuNCs. By coating the co-loaded pSiNP with antibodies targeting each specific cell surface receptor (anti-HER2 and anti-EGFR), we were able to target two types of BCa cells: HER2-positive AT3 and MDA-MB-231 (a triple negative BCa cell line overexpressing EGFR), respectively. We also developed an RF coil that maximises radiation at 1 GHz, inducing efficient heating of AuNCs, while minimising nonspecific heat dissipation. The coil characterisations indicate minimal return loss, thereby maximising radiation power. Upon the application of an external RF stimulus, AuNCs were activated thereby producing heat, which in addition to the action of the drug showed higher therapeutic efficacy than nontargeted or monotherapy counterparts. The in vitro efficiency was assessed in two-dimensional (2D) cancer cells and three-dimensional (3D) BCa spheroids, which are known to closely mimic human solid tumours and form a more clinically relevant model in preclinical studies [43]. This study established the versatility of this pSiNP-based platform, which could be used for personalised medicine by changing the targeting antibody without compromising therapeutic effects in various cancer cells.

## 2. Materials and Methods

### 2.1. Materials and Reagents

Single-crystalline silicon wafers were purchased from Siltronix (Archamps, France). An alkene semicarbazide linker was synthesized, as described in [44]. Another batch was kindly provided by our collaborators from ICGM, Montpellier; an anti-HER2 antibody was provided by a collaborator from CSIRO; Hydrofluoric acid (HF; 49%) was purchased from J. T. Baker (Center Valley, PA, USA). Dr Jacqui McGovern (Institute of Health and Biomedical Innovation, University of Queensland) kindly provided the MDA-MB-231BO cell line. Ethanol, tetrahydrofuran (THF), dimethylformamide (DMF), dichloromethane (DCM), and trifluoroacetic acid (TFA) were purchased from Merck (Sydney, Australia). Didodecyldimethylammonium bromide (DDAB), decanoic acid, tetrabutylammonium borohydride (TBAB), gold (III) chloride trihydrate (HAuCl_4_.3H_2_O), (±)-α-lipoic acid, toluene, and all other chemicals were purchased from Sigma-Aldrich, St. Louis, MO, USA, unless otherwise stated.

### 2.2. Fabrication of pSiNPs

pSiNPs were prepared by anodic electrochemical etching of p-type silicon wafers (resistivity 0.0055–0.001 Ω cm) by periodically etching at 5 mA/cm^2^ for 20 s and 180 mA/cm^2^ for 0.2 s for 1000 cycles in a 3:1 HF (49%): ethanol solution in a wet bench (AMMT GmbH, Frankenthal, Germany). The pSi film obtained was detached by etching at a constant current density of 180 mA/cm^2^ for 60 s in a 1:1 HF (49%): ethanol solution. The detached membrane was immersed in absolute ethanol in a glass vial and sonicated in an ultrasonicator water bath for 24 h to break down the membrane into smaller particles. pSiNPs were size-excluded by centrifugation. First, particles were centrifuged at 2200 rcf for 6 min to remove any microparticles. Next, the supernatant containing nanoparticles was then centrifuged at 21,000 rcf for 10 min, and optimised to retrieve particles sized ~120–140 nm. The nanoparticles were then stored at 4 °C.

### 2.3. Gold Nanocluster (AuNC) Synthesis

AuNCs were obtained by etching gold nanoparticles, as demonstrated by Cheng-An J et al. [45]. Briefly, appropriate amounts of DDAB and decanoic acid were dissolved in toluene to make a stock solution of 100 mM concentration. Gold precursor solution (25 mM) was then prepared by dissolving gold (III) chloride (HAuCl_4_) in the DDAB solution. Typically, 1 mL of freshly prepared TBAB solution (100 mM in 1DDAB stock solution), was mixed with 0.625 mL of decanoic acid stock solution under vigorous stirring. Then, 0.8 mL of gold precursor solution was injected under vigorous stirring leading instantaneously to a dark-red solution of Au nanoparticles. After two hours of stirring, the gold nanoparticles were collected by adding an excess of methanol until a blue-purple cloudy solution was obtained. Free surfactants, reduction agents, and smaller nanoparticles were removed by discarding the supernatants of this solution by centrifugation at 2500 rcf for 30 min. The wet precipitate of Au nanoparticles was then re-dissolved in 2.5 mL DDAB stock solution, yielding a dark blood-red colour. When these nanoparticles were etched, by adding several drops of gold precursor solution under vigorous stirring, the solution colour turned from dark-red colour to a yellowish transparent solution confirming the formation of AuNCs.

### 2.4. Loading and Modification of pSiNPs

#### 2.4.1. Thermal Hydrosilylation of pSiNPs

A 0.1 M solution of tert-butyl-2[allylamino]carbonyl] hydrazine-carboxylate (SC) was prepared in dry tetrahydrofuran (THF) and was added to freshly etched pSiNPs dispersed in THF in a round-bottomed flask. The solutions were passed through nitrogen to avoid particles being oxidised. The reaction was performed at 95 °C overnight under reflux in an N_2_ atmosphere. SC-functionalised particles (pSiNP–SC) were washed with THF and stored at 4 °C.

To remove the protecting group from the SC linker, pSiNP–SC in THF were centrifuged and rinsed in dichloromethane (DCM) and were further resuspended in a solution of DCM and TFA in a ratio of 3:2 (*v*/*v*). The solution was left under agitation at room temperature (RT) and in the dark for 2 h. The deprotected particles (pSiNP–DP) were washed in DCM and subsequently with ethanol.

#### 2.4.2. Loading of Therapeutics

A 2.5 mg/mL solution of camptothecin (CPT) in DMF was incubated with 1 mg of pSiNP–DP under shaking at room temperature overnight (pSiNP–CPT). For AuNCs loading (pSiNP–AuNC) and co-loading AuNCs and CPT (pSiNP–CPT–AuNC), a 1 mg/mL solution of AuNCs was added alone or along with CPT, respectively, and incubated overnight on an orbital shaker protected from light. After loading, the particles were washed in ethanol to remove any free drugs and AuNCs. The particles were then suspended in 1× PBS for further use. Ab was conjugated afterwards to obtain one batch without the targeting moiety and one with Ab (pSiNP–CPT–Ab, pSiNP–AuNC–Ab, and pSiNP–CPT–AuNC–Ab) to compare the effects of actively targeting cancer cells.

#### 2.4.3. Antibody Conjugation

A solution of 0.5 mg/mL Ab (anti-HER 2/anti-EGFR) was prepared in a buffer composed of 20 mM sodium acetate and 0.15 M sodium chloride at pH 5. A 20 mM sodium periodate solution was also prepared in the same buffer at pH 5. Both solutions were mixed in a 1:1 ratio and were left for 30 min at room temperature in the dark under agitation. The oxidation reaction was quenched by adding ethylene glycol (250 µL). Any unreacted sodium periodate was removed using a centrifugal filter (Amicon^®^, Sigma-Aldrich, St. Louis, MO, USA) with a 10 kDa filter, and was washed with 3× PBS. After antibody purification, pSiNPs were incubated with the antibody for 30 min. Any unattached antibody was removed via centrifugation in sterile 1× PBS three times. A nontargeting pSi-based system was used as a control and was prepared using human IgG antibodies in the same way.

### 2.5. Loading and Release Measurements

The loading efficiency and release experiments were carried out by measuring the absorbance intensity of CPT using a UV–Visible (UV–vis) spectrophotometer (Agilent 8453, Agilent Technologies, Santa Clara, CA, USA) at 340 nm. To measure the loading efficiency, a calibration curve was plotted for different concentrations of CPT. Five hundred µg of pSiNPs loaded with CPT (pSiNP–CPT, pSiNP–CPT–Ab, pSiNP–CPT–AuNC, and pSi–CPT–AuNC–Ab) were resuspended in 1 mL of DMF and sonicated for 30 min to remove the CPT from the pSiNP pores. The concentration of CPT in the solution was then measured using UV–Vis and considered the total CPT loading. For AuNC loading and release (pSiNP–AuNC, pSiNP–AuNC–Ab, pSiNP–CPT–AuNC, and pSiNP–CPT–AuNC–Ab), a similar calibration was plotted by recording the fluorescence intensity of AuNCs (excitation @ 400 nm and emission @ 650 nm) using a plate reader (Perkin Elmer Enspire, Waltham, MA, USA).

To determine the release profile, 1 mg of each sample was diluted in 1 mL of cell culture medium (DMEM) and stored at 37 °C for the duration of the experiment. At each time point (0, 0.5, 1, 2, 3, 4, 10, 24, and 48 h), an aliquot of 100 µL of the pSiNP solution was centrifuged for 5 min at 20,000 relative centrifugal force (rcf) to remove the pSiNPs. The CPT and AuNC contents in the obtained supernatant were measured using UV–Vis and fluorescence, respectively, as indicated before.

### 2.6. Dynamic Light Scattering and ζ Potential Measurements

The mean hydrodynamic diameter of pSiNPs and AuNCs and the size distribution and zeta potential (ζ-pot) of pSiNPs were determined with dynamic light scattering (DLS) using a Zetasizer Nano ZS. A scattering angle of 173° and a temperature of 25 °C were used with pSiNPs dispersed in Milli-Q water.

### 2.7. Transmission Electron Microscopy

Freshly etched pSiNPs and AuNCs were imaged using TEM (JEOL JEM-2100F, JEOL, Tokyo, Japan) equipped with a field emission gun. Samples were diluted and deposited on Formvar film-coated copper grids (PST ProSciTEch, Townsville, Australia). Images were acquired at 200 kV accelerating voltage. Particle size and pore sizes were measured using ImageJ.

### 2.8. Fourier Transform Infrared Spectroscopy

A concentrated aliquot of pSiNPs in ethanol and AuNCs in Milli-Q was spotted on an IR crystal and was air-dried. IR spectra were recorded using a Perkin Elmer Frontier IR (Waltham, MA, USA) in attenuated total reflectance infrared (ATR-IR) mode. Spectra were acquired between 650 and 4000 cm^−1^ at a resolution of 4 cm^−1^ for 64 scans.

### 2.9. Determination of pSi Concentration

A 1 mL aliquot of sample was pipetted into a Teflon beaker and heated to partially evaporate the ethanol in the solution. The sample that remained was then digested with 0.2 mL of 48% HF, 2 mL of 69% nitric acid (HNO_3_), and 2 mL of Milli-Q water. Once the reaction had ceased, the solutions were then made up to 50 mL with Milli-Q water in a plastic volumetric flask. The solutions were analysed using the Varian 730-ES axial ICP–OES (Agilent Technologies, Santa Clara, CA, USA). Certified multielement solutions were used to confirm the accuracy of the calibration standard and the method used.

### 2.10. RF Setup

The custom-made electromagnetic radiation generator (Figure S1A) used consisted of a variable radiofrequency generator set to 1 GHz (Wavetek 2500A Signal Generator 0.2 MHz to 1.1 GHz, San Diego, CA, USA) connected to an amplifier (Mini Circuits^®^, New York, NY, USA), which was powered by an external source (XP Power 28 V, 199 W). Voltage and intensity were adjusted to obtain a power of 15 W, which was measured using an in-line wattmeter (Bird Electronic Model 43 RF Wattmeter, accuracy ±5% full scale; average mode; Bird Technologies, Solon, OH, USA). The AuNCs were introduced into the solenoid and exposed to a radiofrequency field. Since AuNCs are paramagnetic in nature [36], when placed inside a solenoid, they produce heat upon the application of RF as a response to the oscillating magnetic field component of the electromagnetic waves [46]. The solenoid was 4 cm in length and 3 cm in diameter, and it was formed by a copper coil (0.57 mm diameter).

A 1 GHz sinusoidal signal was generated by the radiofrequency generator and was amplified to 10 W using a power amplifier. The amplified signal was then applied to a 2-layer coil tuned to resonate at 1 GHz frequency for maximum power transfer. The magnetic flux density generated by a multilayer coil was greater than that in a single-layer coil as magnetic flux density is directly proportional to the number of turns of a coil. Table S1 shows the parameters of the designed coils. The temperature during the experiments was recorded using an infrared camera (FLIR TG165, FLIR Systems, Wilsonville, OR, USA).

### 2.11. Cell Culture

MDA-MB-231 BO and AT3 (ATCC CRL-2375) cells were maintained in DMEM supplemented with 10% FBS and 1% GlutaMAX. Cells were cultured at 37 °C and 5% CO_2_, and experiments were conducted on cells that were passaged at least twice after thawing from the frozen stock. Cells were used until 12 subsequent passages and the new frozen stock was thawed afterwards. Cells were routinely tested negative for mycoplasma contamination by using a PlasmoTest™ Mycoplasma Detection Kit (rep-pt1, Invitrogen, San Diego, CA, USA).

### 2.12. Confocal Microscopy

The active targeting of Ab-coated pSiNPs was confirmed using a confocal laser scanning microscope (Leica TCS SP8, Leica Microsystems, Wetzlar, Germany).

The antibody was labelled using NHS-Cy5 (Thermo Fischer Scientific, Waltham, MA, USA) before attachment to pSiNPs. Twenty µL of a 1 mg/mL solution of Cy5 (in DMSO) was added to 0.5 mg/mL antibody in sodium bicarbonate buffer (100 mM, pH 8) and was left overnight at 4 °C. Any unbound Cy5 was then removed from the antibody solution using a centrifugal filter (Amicon^®^, cut-off of 10 kDa) with PBS 1× until the solution appeared colourless.

For cell preparation, 1 × 10^4^ cells (AT3 or MDA-MB-231 BO) were seeded per chamber in an 8-chambered slide (Thermo Fischer Scientific, Waltham, MA, USA) and allowed to attach overnight. One hundred ug/mL pSiNP–antiEGFR was used to target MDA-MB-231 BO cells, while pSiNP–antiHER2 was used to target AT3 cells. Unmodified pSiNPs and pSiNP–human IgG (human IgG) were used as a nontargeting control. Cells were washed thoroughly with 1× PBS to remove any unbound pSiNPs after 1 h, followed by further 24 h incubation of attached particles with cells. Cells were fixed using 4% PFA for 15 min followed by a further 5 min incubation to permeabilise cells using 0.1% Triton-X-100 in PBS. A staining solution containing Hoechst 33342 [1:5000 dilution; stock-10 mg/mL (Thermo Fischer Scientific, Waltham, MA, USA)] and Phalloidin-TRITC [1:500 dilution; stock-0.2 mg/mL (Sigma-Aldrich, St. Louis, MO, USA)] in 1× PBS was prepared. After washing the cells with 1× PBS, 100 µL of the staining solution was added to each chamber and the slide was left at 37 °C to incubate for 30 min. Finally, the chambers were detached, and a cover slip was mounted on the slide with Prolong TM Diamond Antifade Mount Reagent (Thermo Fischer Scientific, Waltham, MA, USA).

For 3D spheroids, cells were cultured using the forced floating method in a low adhesion polymer coated 96-well plate (Corning, Somerville, MA, USA, ultra-low attachment round bottom). Typically, 100 µL of 10^4^ cells/mL were seeded per well and the spheroids were allowed to grow for 5 days at 37 °C and 5% CO_2_. The size of the spheroids was continuously monitored using brightfield imaging (ZOE Fluorescent Cell Imager, Bio-Rad Laboratories, Sydney, Australia).

Cy5-labelled pSiNPs (pSiNP–Ab) were incubated with the spheroids for 2 h followed by a further 24 h incubation after washing for any unbound particles. The spheroids were rinsed 3× with PBS, transferred to a chamber slide, and fixed in 4% paraformaldehyde (PFA) overnight at RT. After rinsing 3× in PBS to remove any residual PFA, the spheroids were further embedded using an embedding medium (Tissue-Tek^®^, ProSciTech, Sydney, Australia) on dry ice. The frozen blocks obtained were stored at −80 °C or on dry ice until cryo-sectioning was performed.

Eight µm sections were cut and labelled using Prolong TM Diamond Antifade Mount (Thermo Fischer Scientific, Waltham, MA, USA). The sections were further visualized using a confocal microscope.

### 2.13. Flow Cytometry

AT3 and MDA-MD-231 BO cells were seeded in 24-well plates at a cell density of 1 × 10^5^ and were left to attach overnight. Cells were washed with 1× PBS and were incubated with 100 µg/mL pSiNP–anti-HER 2, pSiNP–anti-EGFR and pSiNP–human IgG for 1 h. All Ab were tagged with Cy5 for fluorescence. The cells were washed 3× with PBS to remove any unbound pSiNPs. After 24 h, cells were detached using 100 µL of trypsin-EDTA per well. Once the cells detached, 400 µL of DMEM media was added to the wells and cells were collected in centrifugation tubes. Cell pellets were collected and washed twice using PBS by centrifugation at 200 rcf for 5 min. The cells were further stained with 7-AAD for 5 min on ice and washed in PBS. Cell pellets were finally suspended in FACS buffer (1× PBS with 10% FBS, 2 mM EDTA, and 0.1% NaN_3_) and kept on ice until analysis. Samples were analysed using flow cytometry (BD FACS Canto II, BD Biosciences, Aalst, Belgium) for Cy5 fluorescence. Cellular association percentage was calculated as the number of cells that showed fluorescence when compared to untreated cells. All experiments were performed in triplicate.

### 2.14. Cell Viability

Special miniaturised well plates were made from polydimethylsiloxane (PDMS) to fit in the RF coil for experiments. PDMS (liquid) was mixed with a cross-linking agent (ratio 10:1) and poured into a Petri dish. It was incubated at 80 °C overnight to obtain the chip (PDMS cross-linked). Further chips of the required dimension were cut out, holes were made in the chip and further pressed gently on glass slides to easily hold 100 µL of solution.

Cells were seeded in PDMS wells with a glass cover slide at 1 × 10^4^ cells per well and were allowed to attach overnight. One hundred µg/mL pSiNPs in DMEM media were incubated for each sample variant in triplicate. After 1 h of incubation with particles, cells were washed with 1× PBS and fresh media was added to the wells. After a further 24 h of incubation, one batch of chips was treated using RF for 15 min and the other untreated. For RF treatment, PDMS chips were exposed to 1 GHz electromagnetic radiation (microwave range) and generated using a custom-made electromagnetic generator, as described above. Each treatment was performed for 15 min and the setup was left to cool down for 5 min between each treatment. The cell viability was determined using an ATP-based luminescent cell viability assay (CellTiter-Glo, Promega, Madison, WI, USA), as per the manufacturer’s protocol, and after 24 h of RF treatment for monolayer (Figure S1A) and 3D spheroids (Figure S1B). Each experiment was performed in triplicate and compared to the negative (untreated cells) and positive control (cells exposed to 10% DMSO). Luminescence intensity was measured using a PerkinElmer EnSpire multimode (Waltham, MA, USA) plate reader.

Spheroids were cultured as mentioned previously and were treated with 100 µg/mL pSiNPs as for the 2D cell culture but for 2 h. The spheroids were washed and left for 24 h at 37 °C for particle uptake. Before RF treatment, the spheroids were transferred to the PDMS chips and were exposed to radiation, as above. The spheroids were transferred back to the well plates and the viability assay (3D Cell Titer Glo, Promega, Madison, WI, USA) was performed 24 h after the RF treatment. Similar controls were used for the 2D monolayer cell cultures.

### 2.15. ROS Generation

AT3 cells (2 × 10^5^ cells) were seeded in 8-chamber slides (ibidi^®^, Grafelfing, Germany) and left overnight for attachment. Cells were incubated with pSiNPs (100 µg/mL) with different loads for 1 h. Cells were washed 3× with PBS and left for 24 h for internalisation. One mM ascorbic acid (Sigma-Aldrich, St. Louis, MO, USA) was also used as control [47], wherein it was incubated with cells (preincubated with pSiNP–CPT–Ab) for 1 h prior to fluorescence measurements. The slides were divided into two groups, one with and one without RF radiation. After 24 h, 20 µM 2′-7′dichlorofluorescin diacetate (DCF-DA) (Sigma-Aldrich, St. Louis, MO, USA) was added to each well. RF was applied to one batch for 15 min while the other batch was left at 37 °C. The cells were collected after RF and transferred to a 96-well plate and ROS signals were measured by reading fluorescence on a plate reader (Enspire Multimode, Perkin Elmer) using excitation and emission values 485 nm and 530 nm, respectively. The fold-change in ROS was plotted with untreated cells as control (n = 3).

### 2.16. Statistical Analysis

The data were analysed using GraphPad Prism (9.3.1 for Windows, GraphPad Software, La Jolla, CA, USA). One-way ANOVA for multiple comparisons was used to compare the values between multiple groups and determine statistically significant differences (*p* < 0.05).

## 3. Results and Discussion

### 3.1. Synthesis and Characterisation of pSiNPs and AuNCs

**Figure 1:**
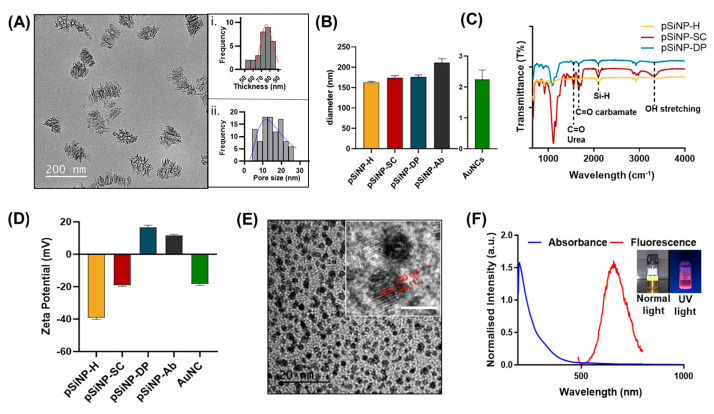
Characterisation of pSiNPs and AuNCs. (**A**) Representative transmission electron microscopy (TEM) image of pSiNPs; insert i: a histogram distribution of nanoparticle thickness (Mean-73 ± 9.0 nm); and insert ii: a histogram distribution of pore size diameter (Mean-14 ± 5.0 nm). (**B**) Dynamic light scattering (DLS) measurements of pSiNPs before and after functionalisation, and AuNCs in Milli-Q. (**C**) FTIR spectra of freshly etched pSiNPs (pSiNP-H), pSiNPs after semicarbazide modification (pSiNP-SC) and after deprotection of the Boc group in the semicarbazide linker (pSiNP-DP). (**D**) ζ-potential of AuNCs and various types of pSiNPs; the term ‘Ab’ here refers to the anti-HER2 antibody. (**E**) TEM image of the AuNCs. Insert: zoomed-in image showing the lattice fringes of AuNCs (Scale bar: 2 nm). (**F**) Absorbance and fluorescence spectra (after excitation at 480 nm) of AuNCs. Insert: AuNCs under visible light and UV light exhibiting red photoluminescence.

pSiNPs were obtained by electrochemical anodisation followed by sonication, as previously described [48]. The nanoparticles were size-excluded via a series of centrifugation processes to obtain plate-shaped (discoidal) nanoparticles of lateral dimensions of 121 ± 10 nm, as confirmed by transmission electron microscopy (TEM) (Figure 1A). The nanoparticle thickness was determined to be 74 ± 9 nm (calculated as the mean thickness of 30 pSiNPs) and the pore size was 14 ± 5 nm (calculated as the mean of 100 randomly selected pores). The histograms were mounted using the Sturges method [49]. The bin-width (W) is obtained from the relationship: W = (Dmax − Dmin)/k, where k = 1 + 3.322 log (N), and N is the number of values. The histogram was further modelled using a log-normal distribution, as shown in Figure 1A(i,ii). The dynamic light scattering (DLS) showed an average particle size of 158 ± 4 nm for freshly etched pSiNPs (pSiNP–H) (Figure 1B). As pSiNPs obtained are discoidal in shape [50], DLS size will vary from the actual size as DLS might not take the contribution of rotational diffusion into account for nonspherical particles [51].

Freshly etched pSiNPs were functionalised with *tert*-butyl2[(allylamino)carbonyl] hydrazine-carboxylate (Boc-protected alkene semicarbazide linker) via thermal hydrosilylation to allow the oriented attachment of an oxidised antibody (Ab) through the Fc (Fragment, crystallisable) region, enhancing the Ab’s site-specific selectivity [52,53]. The hydrosilylation of the Boc-protected semicarbazide linker to pSiNPs (pSiNP–SC) was confirmed using Fourier transform infrared spectroscopy (FTIR) (Figure 1C). The disappearance of the Si–H vibrations at 2100 cm^−1^ indicated that the Si–H groups had been consumed. Furthermore, The IR spectra of pSiNP–SC displayed IR peaks at 1705 cm^−1^ and 1650 cm^−1^ corresponding to C=O stretches from the carbamate and urea groups present in the semicarbazide molecule, respectively, further confirming the success of the reaction. When the Boc protecting groups were removed using trifluoroacetic acid (TFA), the carbamate (C=O) peak disappeared, confirming the deprotection of the Boc group (pSiNP–DP). Moreover, the ζ-potential was monitored at each surface modification stage to corroborate the FTIR spectral analysis (Figure 1D). A negative ζ-potential value was obtained for freshly etched pSiNPs (−40 ± 1 mV), which is some level of oxidation of the reactive Si–H groups. For pSiNP–SC and pSiNP–DP, a ζ-potential of −19 ± 2 mV and 16 ± 1 mV was recorded, respectively. The positive ζ-potential from pSiNP–DP further validated the deprotection of the Boc group, revealing terminal amines. To enable the selective targeting of cancer cells, pSiNP–DP were incubated with periodate-oxidised anti-HER2 (pSiNP–HER2) or anti-EGFR (pSiNP–EGFR) Ab’s. The size of pSiNPs increased to 182 ± 10 nm after modification, consistent with antibody bioconjugation (Figure 1B).

AuNCs were prepared by etching freshly synthesised 5 nm gold nanoparticles [45]. Synthesised dihydrolipoic acid (DHLA)-coated AuNCs were dark yellow with a size of 2.1 ± 0.3 nm, as measured via DLS (Figure 1B) and further confirmed by TEM (Figure 1E). The UV–visible absorbance spectrum showed no plasmon peaks, confirming the absence of gold nanoparticles > 3 nm in diameter, and, thus, that only ultrasmall-sized AuNCs were present [35]. The fluorescence spectra of AuNCs exhibited an emission maximum at 645 nm following excitation at 480 nm (Figure 1F). Accordingly, these AuNCs emitted bright red fluorescence (Figure 1F-insert) when irradiated with ultraviolet light (365 nm).

### 3.2. Loading and Release of Therapeutics

pSiNPs have been established as efficient carriers for therapeutics due to their high drug-loading capacity and controllable drug release through surface functionalisation [54]. Camptothecin (CPT) is a poorly soluble drug that targets and destabilises DNA topoisomerase I in the cell nuclei, inhibiting DNA relegation and thereby causing apoptosis [55]. We have previously demonstrated pSiNPs as effective vectors for CPT delivery with a drug-loading efficiency of 11–15 wt% [15,52]. To further enhance their capacity to load both CPT and AuNCs, we increased the total surface area by fabricating pSiNPs with smaller pore sizes (12 nm) compared to Secret et al. (24 nm) [52].

**Figure 2:**
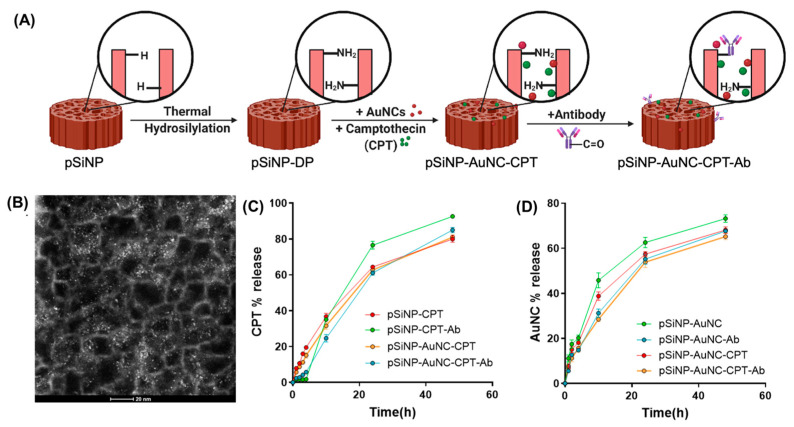
Loading and release of therapeutics. (**A**) Schematic showing modification steps of pSiNPs co-loaded with CPT and AuNCs followed by the attachment of targeting Ab. (**B**) Dark-field TEM image of pSiNP–AuNC, where white dots represent AuNCs. Release kinetics graph of (**C**) CPT and (**D**) AuNCs from pSiNPs with different functionalities. Here, ‘Ab’ refers to the anti-HER2 antibody.

After the functionalisation process, CPT and/or AuNCs were loaded into pSiNPs followed by the coupling of anti-HER2 (Figure 2A). pSiNPs were loaded with either CPT (pSiNP–CPT) or AuNCs (pSiNP–AuNC) or with both (pSiNP–CPT–AuNC). CPT and AuNC loading and release from pSiNPs were quantitatively evaluated by measuring CPT absorbance and the fluorescence of AuNCs. The loading amounts and efficiency of pSiNPs were calculated and displayed in Table 1. Supernatants obtained during the washing before and after Ab attachment confirmed negligible amounts of CPT or AuNCs had been released from the pSiNPs during Ab conjugation. The presence of AuNCs (white dots) within the pSiNP structure was further confirmed using dark-field TEM (Figure 2B).

The release of CPT and AuNCs from pSiNPs with or without Ab (Figure 2C,D) was followed over 48 h in cell culture media. The release of CPT from targeted and non-targeted pSiNPs (Figure 2C) after 4 h was 1.8 ± 2% and 19 ± 1%, respectively, indicating a burst release of the drug from nontargeted pSiNPs. We hypothesise that the conjugation of Ab on the surface of pSiNPs impeded the release of CPT due to partial pore blockage. Similarly, for AuNCs (Figure 2D), over the initial 10 h of incubation, 30 ± 4% was released from pSiNP–AuNC–Ab compared to 45 ± 2% pSiNPs without Ab conjugation, also indicating that the Ab hindered the AuNCs leaking out as fast from the pores. After the same time (10 h), pSiNP–CPT–Ab showed 34 ± 7% CPT release (Figure 2C), which increased to 76 ± 8% over 24 h. Comparatively, CPT release from pSiNP–CPT–AuNC–Ab was slightly retarded and showed a release of 61 ± 3% of total CPT over the same period. After 48 h incubation of pSiNPs co-loaded with AuNCs and CPT (pSiNP–CPT–AuNC–Ab), 65 ± 1% of the AuNCs were released into the media. A sustained release profile helps prevent any premature therapeutics release before the nanoparticles reach the target cells, ensuring the cargo is delivered to the target site [56].

### 3.3. Design and Characterisation of the Radiofrequency (RF) Field Generator

The generation of heat under RF can be attributed to the fact that these AuNCs respond to the field’s oscillating magnetic component due to their paramagnetic nature [46]. Therefore, we designed an RF setup, as shown in Figure S2A, that favours the generation of a magnetic field to maximise the interaction of AuNCs with the 1 GHz electromagnetic field. This was achieved using a multilayered coil that was able to generate a dynamic magnetic field when 1 GHz of alternating current was applied to the coil.

An important parameter to consider when designing this system was to minimise the nonspecific heating induced by the coil, so we ensured that the heating generated stems predominantly from the AuNC–RF interaction. There are three main factors governing the amount of induced heat that is generated by the coil: (i) the amount and the frequency of the magnetic field flux that produces the induced current [57,58], (ii) the properties of AuNCs, including its resistivity [40,59], and (iii) the orientation and exposure duration of AuNCs interacting with magnetic field flux [60,61]. The magnetic field flux (B) and the induced electric field (E) due to the RF coil can be stated by introducing the vector potential A, as indicated in Equation (1);

(1) $$\overset{\to }{B}=\nabla XA$$

From Equation (1), with the assumption of the axisymmetric condition for the coil, A_θ_ is the only component that cannot be zero, which yields

(2) $$\left\{\begin{array}{c} B_{r}=-\frac{\partial A_{\theta }}{\partial z}\\ B_{z}=\frac{1}{r}\frac{\partial (rA_{\theta })}{\partial r} \end{array}\right.$$

where B_r_ and B_z_ are the radial and axial components of the magnetic field flux produced by the coil, and r is the radial distance from the centre of the coil (Figure S2B). The magnetic field in the centre of the coil is uniform at its maximum magnitude, and its direction is along the axis of the coil. As a result, the contribution of the radial magnetic field flux B_r_ can be neglected. By solving A_θ_ in Equation (2) for a coil that is made of multiple circular current loops, B_z_ can be calculated using Equation (3):

(3) $$B_{z}=\frac{a\mu ni}{2\pi }\int_{0}^{\pi }{\left[\frac{\xi (a-\mathrm{rcos}\emptyset )d\emptyset }{\left(r^{2}+a^{2}-2ar\cos\emptyset \right)\sqrt{\xi^{2}+r^{2}+a^{2}-2ar\cos\emptyset }}\right]}_{\xi_{-}}^{\xi_{+}}$$

where *a* is the radius of the coil, *n* is the number of turns per unit length *L*, *i* is the current flowing in the coil, *µ* is permeability, and ξ=z±L/2, where *z* is the axial distance from the centre of the coil (Figure S2B).

From Equation (3), it can be seen that the magnitude of the magnetic flux is directly proportional to the number of turns of a coil, hence an RF coil was designed with a total of 128 turns (Table S1). To maximise the output of the coil at 1 GHz frequency, different core structures, the number of layers of windings, and different dimensions of the core were tested. Table S1 shows the finalised parameters of the coil, which exhibit low return loss at 1 GHz frequency along with high magnetic field strength. The magnetic field flux generated by the RF coil was estimated by both simulation and mathematical models (Figure S3A,B). Using COMSOL Multiphysics and the parameters in Table S1, simulations showed that the highest density of the magnetic field flux was around the copper wire (Figure S3A). However, the samples cannot be placed so close to the coil as they could be affected by the mild heat dissipation from the coil. Moreover, the magnetic field is highly heterogeneous towards the edges of the coil (Figure S3B, red), meaning that not all of the samples would interact with the same magnetic flux density—affecting the heating performance of the AuNCs. Therefore, samples were placed where the magnetic field was more uniform: in the centre of the coil where the flux was between 23.8 and 25.0 mT. To validate these results, the magnetic field flux was also calculated using MATLAB and Equation (3) (Figure S3B, black), matching those observed by the simulation.

To determine the efficiency of the coil, the power that reflects from the coil, the coil return loss, was calculated using Equation (4):

(4) $$RL=-20{log}_{10}\left|S_{11}\right|dB$$

where $S_{11}$ is the input voltage reflection coefficient, which can be measured using a vector network analyser (VNA). The return loss values of the coil at different frequencies are shown in Figure S3C. Usually, return loss values around −10 dB are considered good as they show that less power is reflected [62]. The cause of this power reflection is the impedance mismatch between the source and coil due to improper termination. Using an RF field strength power meter, the coil’s field strength was measured to be 36.5 dBm (4.5 W). Thus, the coil demonstrated a low return loss of −11.1 dBm at 1 GHz (Figure S3C).

### 3.4. Heating Properties of AuNCs under RF at 1 GHz

AuNCs were assessed for their capability in producing localised heat upon the application of an external RF field using our solenoid RF generator (Figure S2A). A solution of AuNCs in PBS was exposed to an RF field (1 GHz, 10 W), and the temperature was recorded against time. The AuNCs solution, starting at 21 °C, reached a temperature of 37 °C after 15 min of RF exposure (Figure S4). Conversely, the temperature of PBS alone only reached 25 °C after 15 min of RF exposure. Therefore, the presence of AuNCs caused a rise in temperature of 16 °C, while without AuNCs, the solution only rose 4 °C due to heat transfer from the coil. After 30 min of RF exposure, the AuNC solution reached temperatures of 48 °C as opposed to the 28 °C obtained for the AuNC-free solution, confirming not only that AuNCs can generate enough heat to be measured as a significant temperature rise in the bulk solution but also that the heat dissipation from the coil is not causing a significant rise in temperature in the buffer solution.

### 3.5. Cellular Association and Uptake of Antibody-Targeted pSiNPs

The ability of targeted pSiNPs to selectively bind to cancer cells was investigated in two different types of BCa cell lines, AT3 and MDA-MB-231-BO. The AT3 cell line is known to overexpress HER2 while MDA-MB-231-BO is a TNBC cell line known to overexpress EGFR [63].

pSiNPs were modified using antibodies targeting the receptor of interest for each cell line—Trastuzumab, a monoclonal antibody specifically targeting HER2 (pSiNP–anti-HER2) [64], and Cetuximab (Erbitux), an anti-EGFR monoclonal antibody (pSiNP–anti-EGFR) [65]. The amount of attached antibody was analysed using a UV absorbance measurement. The concentration of the attached antibody on pSiNPs was 42 ± 2 µg of anti-HER2 Ab per mg of pSiNPs, and 46 ± 1 µg anti-EGFR Ab per mg of pSiNPs.

**Figure 3:**
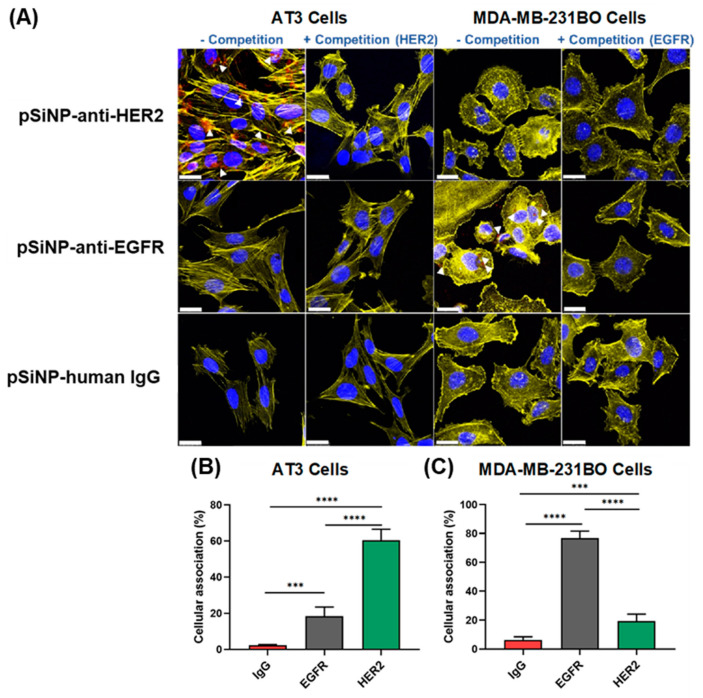
Cellular association of pSiNPs. (**A**) Confocal microscopy images of AT3 and MDA-MB-231-BO cells after competitive inhibition assay. Cells were pretreated with free Ab (anti-HER 2, anti-EGFR, and human IgG) prior to the addition of pSiNPs for the + Competition group. One hundred µg/mL pSiNPs attached with different Cy5-labelled Ab were incubated with cells for 1 h and washed before imaging. The blue, yellow, and red stains represent the nucleus, cytoskeleton, and Cy5-labelled Ab-attached pSiNPs, respectively (Scale bar: 50 µm). Flow cytometry results for the cellular association of Ab-attached pSiNPs (human IgG, anti-EGFR, and anti-HER2) against (**B**) AT3 cells and (**C**) MDA-MB-231BO cells. Data shown as mean ± SD (n = 3, *** *p* ≤ 0.001, and **** *p* ≤ 0.0001).

The cellular association of pSiNP–anti-HER2 and pSiNP–anti-EGFR was evaluated against the two breast cancer cell lines using confocal microscopy and flow cytometry to demonstrate the cell specificity of our nanoparticles (Figure 3). The antibodies (anti-HER2 and anti-EGFR) were tagged with Cy5 prior to the attachment to pSiNPs to allow fluorescence detection. Confocal microscopy images (Figure 3A) qualitatively confirmed the binding of Cy5-labeled pSiNP–anti-HER2 to AT3 cells, which are HER2-positive. In contrast, no binding of pSiNP–anti-HER2 was observed when AT3 cells were preincubated with free anti-HER2 antibodies, attributable to the active competition for HER2 receptors on the cell surface. The same pattern of results was observed when MDA-MB-231-BO cells (EGFR-positive) were incubated with Cy5-labeled pSiNP–anti-EGFR in the presence or absence of a free anti-EGFR antibody (Figure 3A). As controls, pSiNP–anti-HER2 and pSiNP–anti-EGFR were incubated with the opposite cell line where cell surface receptors were not overexpressed (e.g., MDA-MB-231-BO incubated with pSiNP–anti-HER2 as a negative control). Confocal microscopy images showed that when cross-incubated with opposing cell lines, no visible Cy5-labeled pSiNPs could be detected. Similarly, cells incubated with pSiNPs functionalised with a nontargeting IgG human monoclonal antibody showed no visible fluorescence. Taken together, this suggests that cellular association was receptor-mediated as no visible particle association was observed when (i) in competition with free antibody, (ii) incubated with cells that do not overexpress the corresponding receptor, and (iii) when the antibody on the surface of the pSiNP is nonspecific. To further corroborate this, we quantified the cellular association via flow cytometry (Figure 3 and Figure S5). After incubating pSiNPs with AT3 cells for 1 h, 1.9 ± 0.2%, 15.1 ± 3%, and 57.2 ± 6% of cells had associated with pSiNP–human IgG, pSiNP–anti-EGFR, and pSiNP–anti-HER2, respectively (Figure 3B). When incubating pSiNP with MDA-MB-231-BO cells for 1 h, 1.3 ± 1%, 72 ± 3%, and 15.2 ± 3% of cells had associated with pSiNP–human IgG, pSiNP–anti-EGFR, and pSiNP–anti-HER2, respectively (Figure 3C). The obtained results demonstrate that the antibody-displaying pSiNPs can selectively target specific cells based on the receptors present on the cell surface. Previous literature suggests that pSiNPs are readily endocytosed after being actively recognised by cell surface receptors [66].

**Figure 4:**
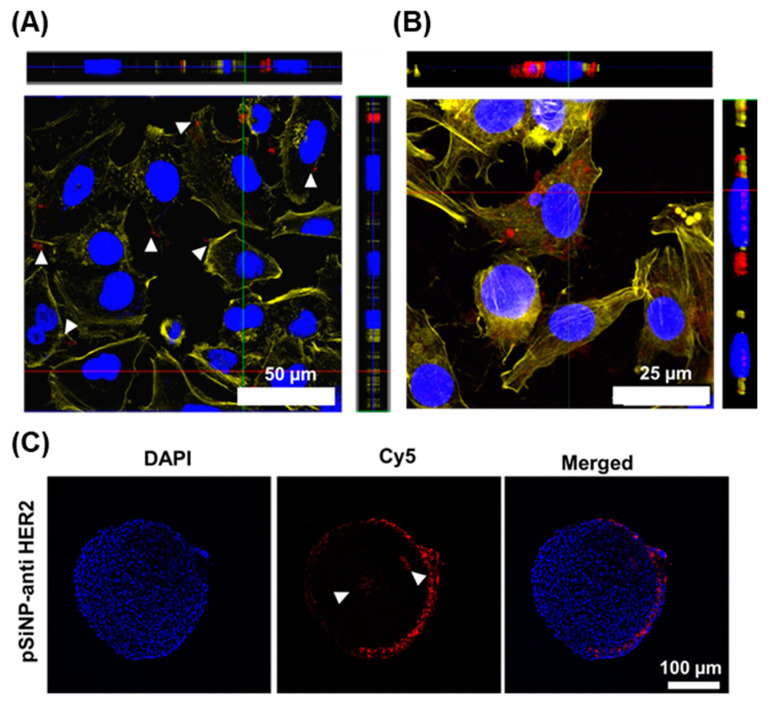
Particle internalisation in AT3 cells. (**A**) z-stack confocal images showing the uptake of Cy5-labelled pSiNP–Ab (shown in red colour in the left image; scale bar: 50 μm), (**B**) AuNCs (after incubation of cells with unlabelled pSiNP–AuNC–Ab) (AuNCs shown in red colour in the right image; scale bar: 25 μm) by AT3 cells (blue: nucleus, yellow: cytoskeleton), and (**C**) Confocal images of the cross-section of AT3 3D spheroids after incubation with Cy5-labelled pSiNP–Ab (blue: nucleus, red: pSiNPs). Here, ‘Ab’ refers to the anti-HER2 antibody. Scale bar: 100 μm.

pSiNPs that bound to the cells selectively were further left under incubation to study their cell uptake. We confirmed the internalisation of pSiNPs in AT3 cells by z-stacking with confocal microscopy. Cy5-labelled anti-HER2-functionalised pSiNPs (pSiNP–Ab) were incubated with AT3 cells for 1 h. After 1 h, the cells were washed with PBS to remove unbound pSiNPs and were further incubated for 24 h. The confocal z-stacks confirmed pSiNP uptake inside the cells (Figure 4A). Furthermore, AuNCs loaded pSiNP–Ab (the anti-HER2 antibody was not labelled with Cy5 here) were also incubated with AT3 cells, as mentioned above. AuNCs can be seen to be concentrated inside the cells (Figure 4B), which helps ensure that the heat source comes from the inside of the cell (inside-out heat transfer) compared to traditional hyperthermia where the source is located on the outside [67], thereby minimising harm to healthy tissues.

Compared to the traditional two-dimensional cell culture comprising a single layer of cells, three-dimensional spheroids aim to better mimic the in vivo tumour characteristics with respect to recapitulating tumour growth kinetics and signalling pathways [68]. Spheroids are known to retain certain extracellular matrix components as well as hypoxic and proliferative gradients similar to the conditions of poorly vascularised regions of solid tumours [69].

The penetration capability of antibody-coated pSiNPs into a cellular 3D structure was evaluated using AT3 spheroids. Spheroids of ~500 µm in diameter were incubated with pSiNP–Ab for 2 h and left for a further 24 h incubation after washing off any unbound nanoparticles. Spheroids were fixed, embedded, and sliced by cryosection before imaging via confocal microscopy (Figure 4C). The images show the presence of pSiNPs mainly on the periphery of the spheroids, but the Cy5 fluorescence signal was also detected in its core, demonstrating the capacity of pSiNPs to penetrate a 3D environment.

### 3.6. Cell Viability

We examined the therapeutic potential of combined therapy using pSiNPs in vitro. AT3 BCa cells were seeded on a customised 8-well chip made from polydimethylsiloxane (PDMS) mounted on a glass slide. To optimise RF exposure time, cells were incubated with 100 µg/mL pSiNP–Ab loaded with AuNCs and both CPT and AuNCs. Cells were also incubated with only pSiNP–Ab and AuNCs as controls. After 1 h of incubation, the cells were washed with 1× PBS and were further incubated for 24 h in fresh media. Cells were then exposed to RF for different times (10–30 min). The percentage of viable cells was confirmed after 24 h of the RF treatment using a luminescence-based assay. The temperature in the wells was also recorded using an infrared camera at all time points. Cells alone or cells incubated with pSiNP–Ab or bare AuNCs (Figure S6) remained healthy after 20 min of RF exposure. However, after 25 min of RF exposure, there is an overall decrease in cell viability suspected due to the generation of heat from the coil increasing the temperature of the bulk solution above 43 °C. Meanwhile, cells treated with pSiNP–AuNC–Ab showed 68 ± 2% viability within 10 min, which decreased to 56 ± 1% after 15 min of RF exposure. Furthermore, reduced cell viability was observed for cells treated with pSiNP–CPT–AuNC–Ab within the same time intervals. Therefore, we selected a 15 min RF exposure time for further experiments to avoid any cytotoxicity due to excess heat generation.

**Figure 5:**
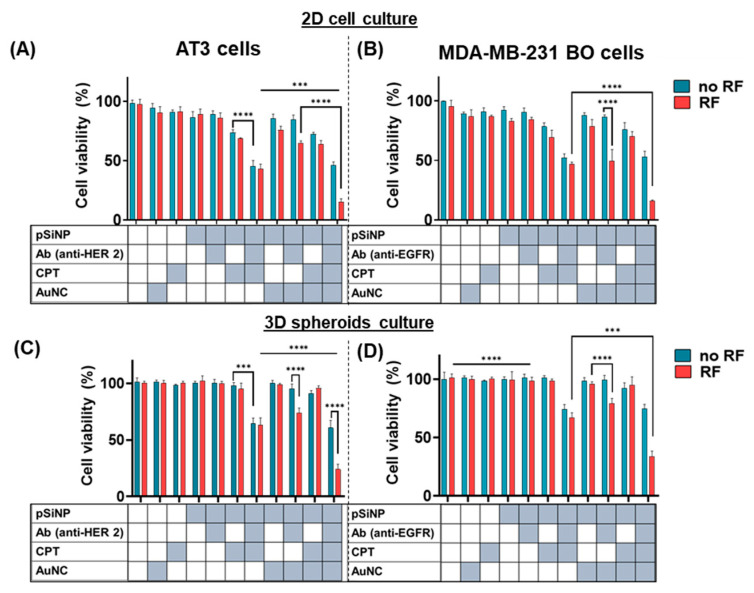
Cell viability upon nanoparticle treatment. Cell viability of AT3 and MDA-MB-231-BO cells in 2D cell culture (**A**,**B**) and 3D cell spheroids (**C**,**D**) after exposure to 100 µg/mL pSiNPs with different conditions with and without RF exposure of 15 min (as per time optimization study shown in Figure S6). Data are shown as a mean ± SD (n = 3, *** *p* ≤ 0.001, and **** *p* ≤ 0.0001).

The cytotoxic effects of the combination of treatments were compared to single therapies. AT3 cells were seeded in an 8-well PDMS chip and treated with pSiNPs with different cargoes for 1 h, as above. The cells were then washed to remove any unbound pSiNPs and were incubated overnight for 24 h. Then, half of the batch was exposed to RF for 15 min, while the other half was left unexposed. The cell viability results (Figure 5A) show that pSiNP–Ab (anti-HER2) were biocompatible and nontoxic to cells with or without RF exposure. We observed 78 ± 4% cell viability when CPT is delivered to cells via nontargeted pSiNPs (pSiNP–CPT), which further decreased to 50 ± 5% when antibody-attached pSiNPs were used (pSiNP–CPT–Ab). Meanwhile, negligible cytotoxicity was observed with free CPT (~40 µg/mL matching the concentration loaded into the pSiNPs), confirming that the antibody-coated pSiNPs are efficient carriers for the successful cellular delivery of CPT. Exposing AT3 cells to hyperthermia alone, that is, AuNCs loaded into antibody-coated pSiNPs (pSiNP–AuNC–Ab) showed no toxic effects on cells until RF radiation was applied, which decreased the cell viability to 76 ± 10%. The application of both therapies in combination, using targeted pSiNPs (pSiNP–CPT–AuNC–Ab), led to a 48 ± 7% cell viability without RF (similar to pSiNP–CPT–Ab). Upon RF exposure, the viability of AT3 cells incubated with pSiNP–CPT–AuNC–Ab drastically decreased to 16 ± 2%. This confirms the synergy between the delivered drug and applied localised heat, resulting in an enhanced therapeutic effect.

We used another adherent cell line to substantiate the results and further explore the versatility of this platform. An aggressive TNBC (cell line MDA-MB-231-BO) was used. In this case, pSiNPs were modified using an anti-EGFR antibody as we have shown their high cellular association before (Figure 3C). The cell-killing efficiency was found to be comparable to AT3 cells (Figure 5B), wherein targeted pSiNPs with both CPT and AuNCs in combination, after treatment with RF radiation, exerted strong cytotoxic effects. The results establish the use of the current system for personalised medicine, since simply by manipulating the targeting Ab on pSiNPs, similar therapeutic efficiency can be achieved in different cancer cell types.

To further confirm the efficacy of the pSiNPs in a more physiologically relevant environment, we used AT3 and MDA-MB-231 BO 3D spheroids after 5 days of culture (Figure S1). A luminescence-based viability assay kit was used to assess the capabilities of the targeted pSiNPs to deliver the combination of therapies. An initial optimisation experiment was performed with different concentrations of anti-HER2-attached pSiNPs (pSiNP–Ab) (50, 100, and 200 µg/mL) incubated with AT3 spheroids at time points of 1 h and 2 h to determine the optimal therapeutic window (Figure S6B). The cell viability results clearly showed that a similar concentration of pSiNPs for 2D cells (100 µg/mL) could be used for spheroid experiments. However, particle incubation for 2 h correlated better with the 2D culture results. 

Figure 5C,D show that the system’s overall efficacy developed even in complex cultures, such as spheroids, and had a similar trend in cell-killing efficiency as in 2D cell cultures. The treatment of both spheroid types AT3 and MDA-MB-231-BO with free drug, free AuNCs, and nontargeted pSiNP loaded with CPT and/or AuNCs showed hardly any effect on cell viability, even after RF exposure. On the other hand, actively delivered CPT (pSiNP–CPT–Ab) showed a cell viability of 62 ± 4% in AT3 spheroids and 66 ± 5% in MDA-MB-231-BO spheroids under no RF. Similar cell-killing efficiency was observed when targeted pSiNPs co-loaded with CPT and AuNCs (pSiNP–CPT–AuNC–Ab) without RF exposure. A significant decrease in cell viability was observed when RF was applied to pSiNP–CPT–AuNC–Ab-treated spheroids. We observed 24 ± 6% cell viability for cells in AT3 spheroids, and 38 ± 4% for cells in MDA-MB-231-BO spheroids. This slight difference in viabilities may be due to the more aggressive nature of TNBC cells compared to AT3 cells. Overall, we observe that this platform offers a versatile approach to target and kill different BCa cell types with high efficiency. The results establish that this system could be used in personalised medicine, wherein the targeting antibody could be tailored based on the cancer subtype while maintaining the cell-killing efficacy of the system. 

Considering the higher toxicity of free drugs to healthy tissues due to nonspecific distribution and uncontrolled diffusion rates, therapeutics-loaded pSiNPs would be preferable for use in combination therapy. We have previously reported cell viability of 13 ± 3% when MDA-MB-231 BO cells were treated with pSiNP–CPT–Ab (anti-EGFR) after 60 h of incubation [15]. We were able to achieve a similar therapeutic effect with a 24 h treatment by adding RF-mediated heat to CPT. Furthermore, it must be noted that the amount of CPT co-loaded with AuNCs into pSiNPs was about 8% less than pSiNPs loaded with CPT alone, but the therapeutic effect was 3-fold more due to the addition of hyperthermia. Therefore, we established that the uptake of CPT and AuNCs by cells followed by the RF treatment induced a therapeutically relevant synergistic effect compared to only drugs or exposure to hyperthermia individually. 

### 3.7. ROS Production in Cells

Increased ROS in cancer cells targets proteins, lipids, and DNA, increasing the cell-killing rate [70]. Here, we used 2′,7′dichlorodihydrofluorescein diacetate (DCFH-DA) to measure the redox state of cells. DCFH-DA is a cell-permeable, nonfluorescent precursor of dichlorofluorescein (DCF) and can be used as an intracellular probe for oxidative stress [71]. Anticancer agents, including CPT, can increase cellular ROS levels in cells [72]. Increased ROS generation was observed for AT3 cells incubated with pSiNP–CPT using DCFH-DA at different time intervals (0 h, 4 h, 10 h, and 24 h) (Figure S7A). The figure indicates an increase in ROS generation over 24 h after treatment with CPT-loaded pSiNPs, with a 2-fold increase in ROS after 24 h. The addition of an antioxidant such as ascorbic acid shows a complete inhibition of increased ROS signals in cells.

Similarly, ROS generation was measured after cells were incubated with pSiNP–AuNC–Ab in the absence and presence of RF. Figure S7B shows that the ROS levels in cells treated with pSiNP–AuNC–Ab were found to be maximum right after RF exposure (within 0.5 h), and subsequently decreased within 4 h after RF. However, the results indicated no ROS generation in cells without RF. Furthermore, pSiNP–AuNC–Ab-incubated cells exposed to 15 min heating (41 °C) instead of RF showed a negligible increase in ROS generation. Therefore, we concluded that the effect of cytotoxicity by ROS generation using combined therapy can be expected to be strongest right after the RF exposure and that the effect dissipates with time.

**Figure 6:**
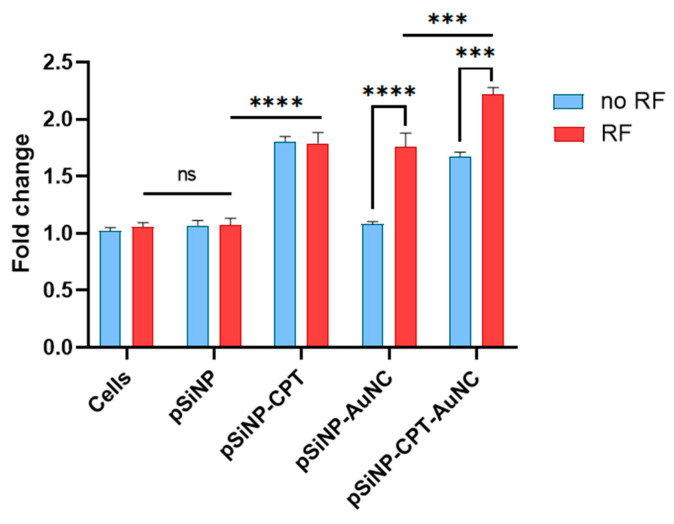
ROS generation. ROS generation in AT3 cells after incubation with 100 µg/mL pSiNPs (pSiNP–Ab, pSiNP–CPT–Ab, pSiNP–AuNC–Ab, pSiNP–AuNC–CPT–Ab) with and without the application of RF. Here, ‘Ab’ refers to the anti-HER2 antibody. (n = 3, *** *p* ≤ 0.001, and **** *p* ≤ 0.0001, ns = not significant).

In a further assessment of the effect of combined therapy on ROS generation in AT3 cells, we observed that the exposure of cells to pSiNPs alone (50 µg/mL) did not result in any ROS generation (Figure 6). As expected, treatment with CPT-loaded pSiNPs (pSiNP–CPT–Ab) showed a 0.8-fold increase in the production of ROS compared to untreated cells. Furthermore, AuNC-loaded pSiNPs (pSiNP–AuNC–Ab) had no impact on ROS production in the absence of RF, while ROS production increased by 0.7-fold as RF was applied, presumably due to the generation of hyperthermia from the activated AuNCs. It is to be noted that the concentration of CPT in pSiNPs loaded with both CPT and AuNCs was 8% (*w*/*w*) lower compared to when just CPT was loaded. This might explain the ROS levels in cells treated with pSiNP–CPT–AuNC–Ab being slightly lower than when cells were treated with pSiNP–CPT–Ab without RF exposure. Interestingly, the combined treatment of cells with CPT and AuNCs co-loaded pSiNPs (pSiNP–CPT–AuNC–Ab) and exposure to RF led to a 1.3-fold increased production of ROS in comparison to the control group. These results, along with the cells viability study (Figure 5A), suggest that the generation of ROS in cells is one of the mechanisms causing higher cell death for combined therapy. The ROS production by AuNCs upon RF may dissipate within 2 h (Figure S7D), but the initial ROS boost may trigger further apoptotic events, which, along with the cytotoxic effects of CPT, lead to higher cell-killing efficiency (Figure S7B) for the combination therapy compared to the monotherapy.

### 3.8. Cell Growth after Treatment in 3D Spheroids

**Figure 7:**
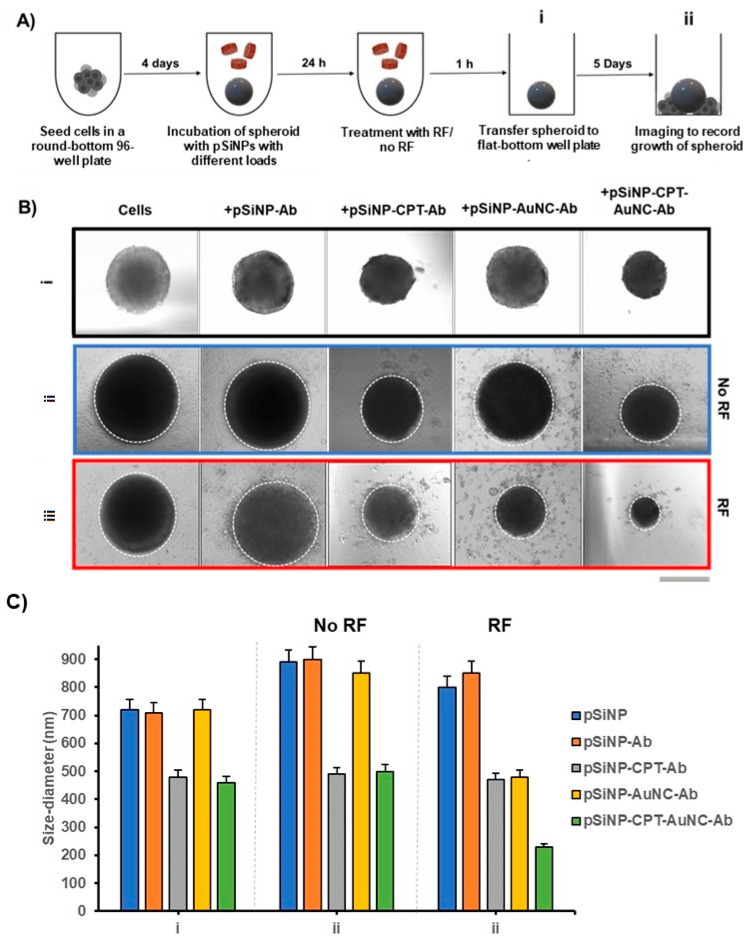
Spheroid response to treatment with pSiNPs with different payloads. (**A**) Schematic illustration of the treatment of AT3 3D spheroids with 100 μg/mL pSiNPs with and without RF treatment followed by imaging to observe spheroids growth and proliferation. (**B**) Brightfield images of AT3 spheroids treated with pSiNPs with different payloads right after they were transferred to flat-bottom plates (black box, (i)) and after 5 days of treatment (blue box (ii) show spheroids that did not receive RF exposure and red box (iii) shows spheroids that received RF treatment for 15 min). The dotted circle denotes the spheroid circumference and the cells beyond that represent the proliferative capacity of cells on a flat surface. (**C**) Change in the size of spheroids after treatment (i) and 5 days after treatment (ii: no RF and iii: RF). Here, the abbreviation ‘Ab’ refers to the anti-HER2 antibody, scale bar: 500 μm.

BCa cells have an accelerated cell division process, invading surrounding tissues and leading to metastasis and the formation of new tumours in other areas of the body. It is essential to ensure the inhibition of cell proliferation to achieve high therapeutic efficiency. We assessed the effect on cell growth in AT3 3D spheroids after treatment with pSiNPs loaded with variable payloads with and without exposure to RF. Figure 7A shows the experimental process to observe changes in the growth rate of spheroids after treatment. The round-bottom well plate format offers low cell attachment, confining the physical space, and promoting the formation of spheroids. Once these spheroids were transferred to flat-bottom plates, the cells attach to the plate surface and continue to proliferate as a spheroid. To understand the impact of the combination treatment on the growth and proliferation of these 3D cell aggregates, we transferred AT3 spheroids once they grew to about 500 µm (diameter) to flat-bottom plates after treating them with pSiNPs with different payloads, with or without RF radiation (Figure 7B). When no treatment was given, the spheroids continued to grow in diameter and showed cell proliferation on the well plate surface. We further observed that spheroids treated with pSiNP–CPT–Ab and pSiNP–CPT–AuNC–Ab without RF exposure were smaller in size compared to spheroids not treated with CPT (24 h post-treatment). This suggests that the proliferative capacity of AT3 cells was reduced after exposure to CPT. However, some viable cells continue to attach to the well plate. Similarly, spheroids treated with pSiNP–AuNC–Ab and exposed to RF showed reduced proliferation and attachment to the surface of the plate for up to 5 days. In contrast, spheroids treated in combination (pSiNP–CPT–AuNC–Ab with RF) showed no proliferation and growth, and shrank in size drastically within 5 days, showing no signs of viability. The images also correlate with the in vitro cell viability assay for spheroids (Figure 5C), wherein the combination treatment showed the highest efficacy in killing cancer cells compared to treatment with pSiNP–CPT–Ab and pSiNP–AuNC–Ab after RF exposure.

## 4. Conclusions

In summary, we developed a pSiNP-based anticancer platform, which enabled hyperthermia in combination with chemotherapy to enhance overall therapeutic efficacy. These Ab-conjugated pSiNPs could (i) actively target corresponding receptor sites on BC cells selectively, (ii) internalise in cells and release the drug and AuNCs, and (iii) produce heat on external stimulation with RF radiation by activating AuNCs. The results concluded higher therapeutic efficacy of the system, which was attributed to not only the successful combination of two therapies but also the fact that a lesser concentration of anticancer drugs was required to kill cancer cells effectively. The platform shows versatility, indicating a potential for use in personalised medicine, wherein the nanocarrier could be used to target different types of cancer cells by modifying them corresponding to the receptors present on target cells. Furthermore, the enhanced amount of ROS in cells after combination therapy indicates ROS generation as a mechanism behind the high efficacy of the system.

This study confirms the potential of applying combined therapies using targeted nanomedicines to treat cancer. This in vitro examination warrants further examination in vivo to help both understand the cell death mechanism induced by the system and accelerate its translation into the clinic.

## Figures and Tables

**Table 1:** Loading efficiency of therapeutics (n = 3).

Sample *	CPT (%w)	Au (%w)
pSiNP	0	0
pSiNP–CPT	41.5 ± 0.5	0
pSiNP–CPT–Ab	39.8 ± 0.2	0
pSiNP–AuNC	0	9.1 ± 0.1
pSiNP–AuNC–Ab	0	8.8 ± 1
pSiNP–CPT–AuNC	33.9 ± 0.3	8.4 ± 0.2
pSiNP–CPT–AuNC–Ab	31.1 ± 0.1	8.5 ± 0.4

* Here, ‘Ab’ refers to the anti-HER2 antibody.

## Data Availability

The authors declare that the data supporting the findings of this study are available within the paper and the supplementary information, or from the corresponding authors upon request.

## Supplementary Materials

The following supporting information can be downloaded at: https://www.mdpi.com/article/10.3390/pharmaceutics15051389/s1, Figure S1: Schematic showing the experimental setup for cell viability assay: (A) for monolayer cells and (B) for 3D spheroids. X in pSiNP–X refers to different loads and functionalities used for the experiments. Figure S2: (A) Customised setup to generate RF radiation in the microwave field (1 GHz). (B) Schematic showing parameters for the coil used for RF generation. Table S1: Design parameters for the RF coil. Figure S3: (A) Simulation of the RF coil in (i) the X–Y plane, (ii) the Y–Z plane, (iii) the X–Y plane, and (iv) the distribution of the magnetic field flux lines of the RF coil. (B) Estimation of the generated magnetic field flux by mathematical (red) and simulation (black) methods using MATLAB and COMSOL Multiphysics, respectively. (C) Measurement of the return loss of the coil using a vector network analyser (VNA). Figure S4: Heating of AuNCs in PBS compared to only PBS when placed inside RF coil for different time points (n = 3). The temperature was measured using an IR camera. Figure S5: Gating used for flow cytometry analysis of HER 2 and EGFR expression in AT3 and MDA-MB-231 BO cells. Figure S6: (A) Cell viability of AT3 cells upon exposure to RF for the time indicated on the X-axis after treatment with AuNCs alone and 100 µg/mL pSiNP–Ab with different loads, and corresponding temperature in the well after the indicated RF exposure time. (B) Cell viability of AT3 spheroids with different concentrations of pSiNPs with different loads to optimise pSiNP concentration and time of incubation (No pSiNP were added to the control group and for just CPT group, the CPT concentration equivalent to the amount loaded in respective pSiNP concentration was added to the cells). Figure S7: (A) Schematic for the experimental flow for determination of ROS generation in AT3 cells by pSiNP–CPT–Ab; (B) ROS generation in AT3 cells after 1 h incubation with 100 µg/mL pSiNP–CPT–Ab; Fluorescence signals for ROS were recorded after 0 h, 4 h, 10 h, and 24 h; (C) schematic for the experimental flow for the determination of ROS generation in AT3 cells by pSiNP–AuNC–Ab nanoparticles; (D) ROS generation in cells treated with 100 µg/mL pSiNP–AuNC–Ab after 15 min RF exposure (fluorescence recorded at 0.5 h, 2 h, and 4h after cells were treated with RF).
