# Supplementary material for: Combination of Chemotherapy and Mild Hyperthermia Using Targeted Nanoparticles: A Potential Treatment Modality for Breast Cancer
Source: Pharmaceutics. 2023 Apr 30;15(5):1389. doi: 10.3390/pharmaceutics15051389 (PMC10222438; doi:10.3390/pharmaceutics15051389)
Supplement: Supplementary file 1 [file pharmaceutics-15-01389-s001.zip › pharmaceutics-2257626-supplementary.pdf]

Supplementary Materials: Combination of chemotherapy and mild hyperthermia using targeted nanoparticles: a potential treatment modality for breast cancer

Ishdeep Kaur [1], Terence Tieu [1], Veerasikku G. Deepagan [1], Muhammad A. Ali [2], Fahad Alsunaydih [3], David Rudd [1], Maliheh A. Moghaddam [4], Laure Bourgeois [5], Timothy E. Adams [6], Kristofer J. Thurecht [7], Mehmet Yuce [2], Anna Cifuentes-Rius [1,*], Nicolas H. Voelcker [1,8*]

[1] Monash Institute of Pharmacy and Pharmaceutical Sciences, Monash University, 381, Royal Parade, Parkville-3052, Victoria, Australia;, Ishdeep.kaur91@gmail.com (I.K.), Terence.tieu@monash.edu (T.T.), gopal.d@wehi.edu.au (V.G.D.), David.rudd@monash.edu (D.R.).
[2] Department of Electrical and Computer Systems Engineering, Monash University, Clayton Campus, Clayton- 3168, Victoria, Australia; Muhammad.ali2@monash.edu (M.A.A.), mehmet.yuce@monash.edu (M.Y.)
[3] Department of Electrical Engineering, College of Engineering, Qassim University, Unaizah 56452, Saudi Arabia; f.alsunaydih@uq.edu.sa
[4] Centre of Polymer Systems, Tomas Bata University, Zlin, Czech Republic, 5678; m_amini_64@yahoo.com
[5] Monash Centre for Electron Microscopy, Monash University, Clayton Campus, Clayton-3168, Victoria, Australia; Laure.bourgeois@monash.edu
[6] Commonwealth Scientific and Industrial Research Organization (CSIRO), 343, Royal Parade, Parkville Victoria 3052; tim.adams@csiro.au
[7] Australian Institute for Bioengineering and Nanotechnology (AIBN), Corner College and Cooper Rds
The University of Queensland, Brisbane Qld 4072, Australia; k.thurecht@uq.edu.au
[8] Melbourne Centre for Nanofabrication, Victorian Node of the Australian National Fabrication Facility, Clayton, Victoria, Australia
* Correspondence: anna.cifuentesrius@monash.edu (A.C.R.), Nicolas.voelcker@monash.edu (N.H.V.)

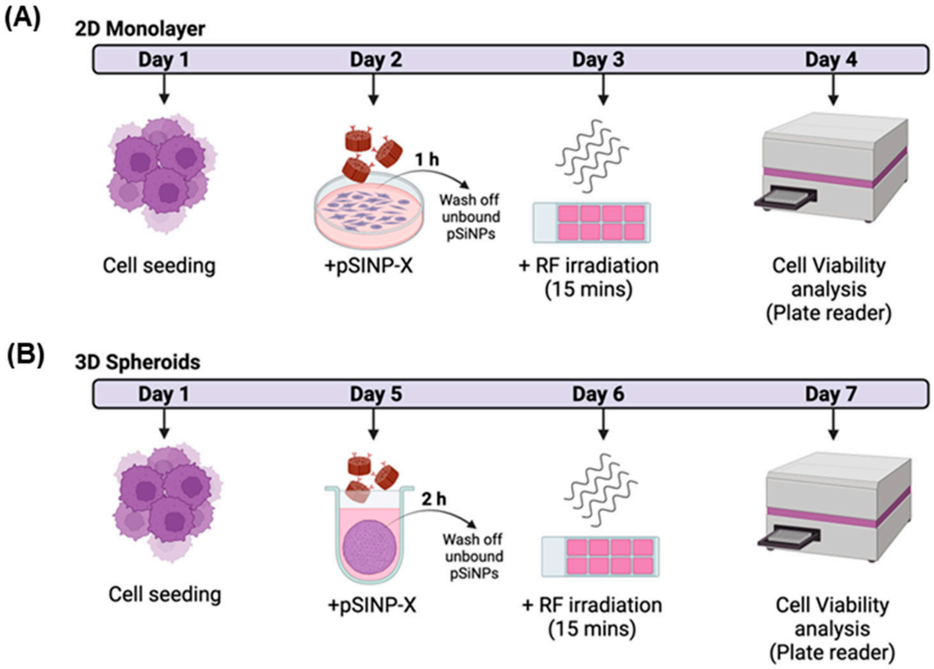

Figure S1. Schematic showing the experimental setup for cell viability assay A) for monolayer cells and B) for 3D spheroids. X in pSiNP-X refers to different loads and functionalities used for the experiments.

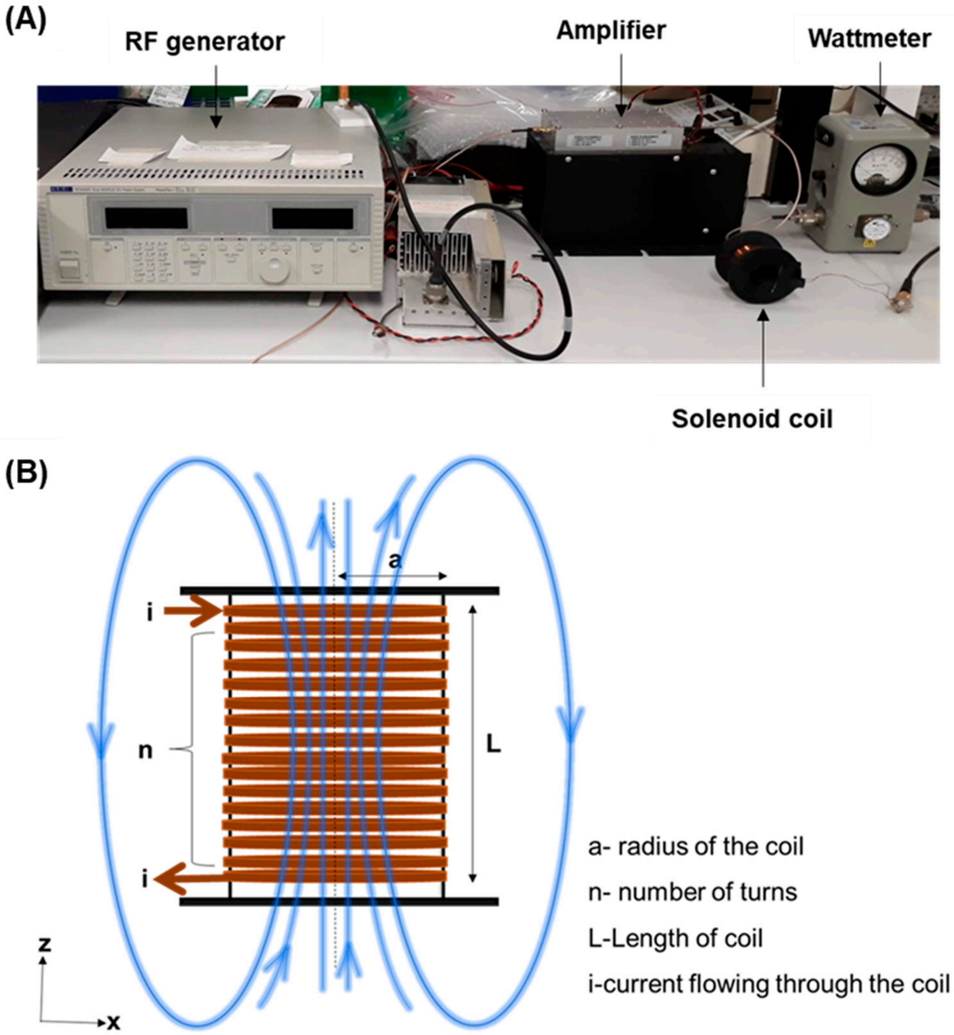

Figure S2. A) Customised setup to generate RF radiation in the microwave field (1 GHz). B) Schematic showing parameters for coil used for RF generation.

Table S1. Design parameters for the RF coil.

Parameter	Value
**Core diameter**	30 mm
**Core length**	40 mm
**Wire diameter**	0.57 mm
**Number of layers**	2
**Number of turns per layer**	64
**Core material**	ABS

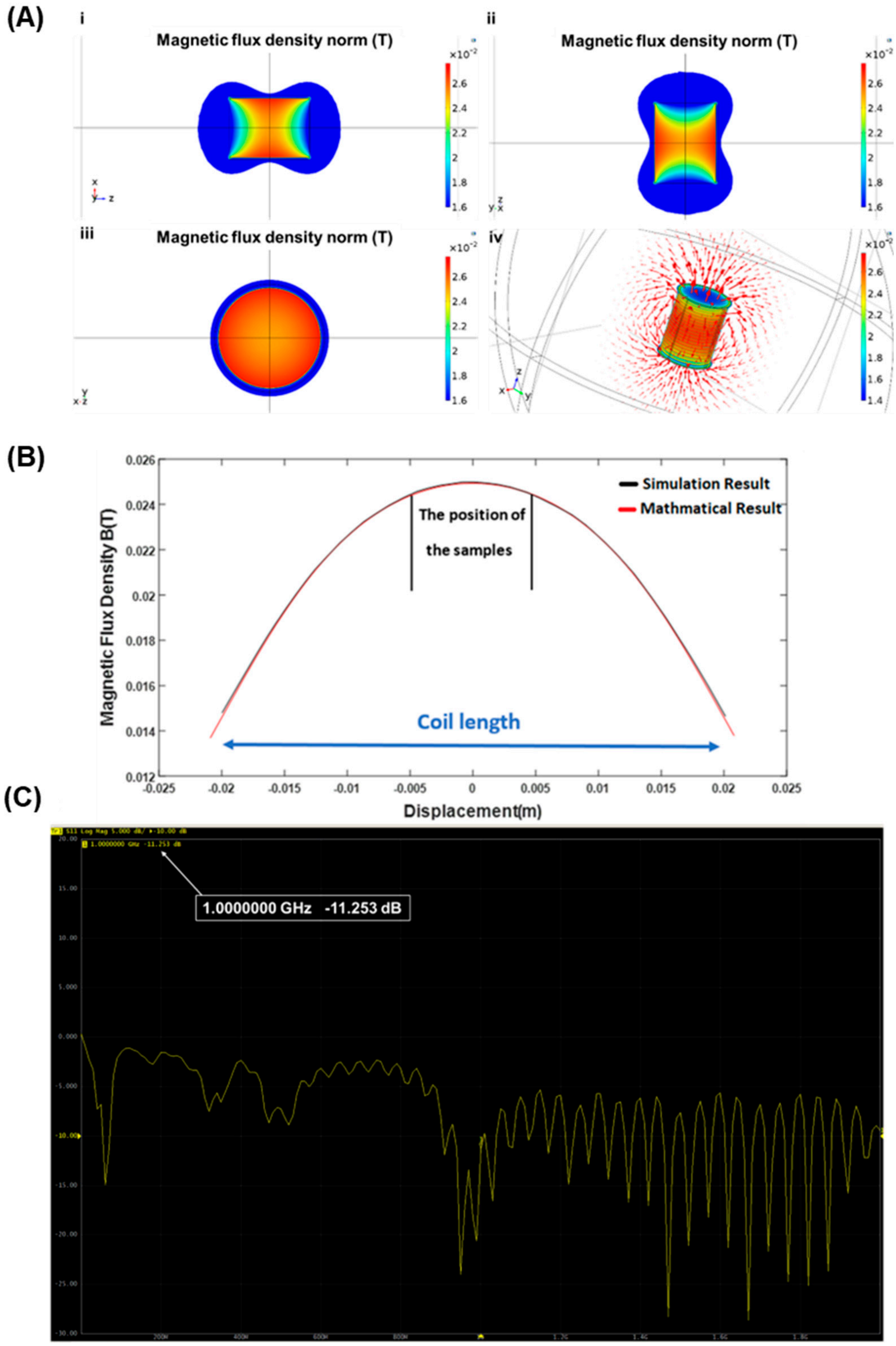

Figure S3. A) Simulation of the RF coil at i) x-y plane, ii) y-z plane, iii) x-y plane and iv) the distribution of the magnetic field flux lines of the RF coil. B) Estimation of the generated magnetic field flux by mathematical (red) and simulation (black) methods, using MATLAB and COMSOL Multiphysics, respectively. C) Measurement of the return loss of the coil using a Vector Network Analyser (VNA).

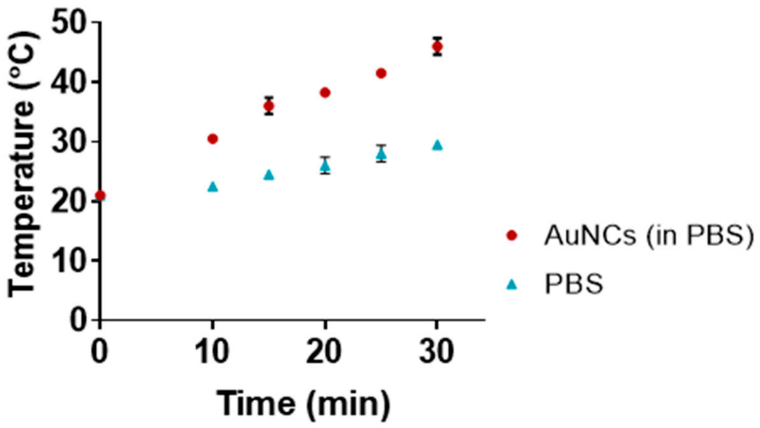

Figure S4. Heating of AuNCs in PBS compared to only PBS when placed inside RF coil for different time points (n=3). Temperature was measured using an IR camera.

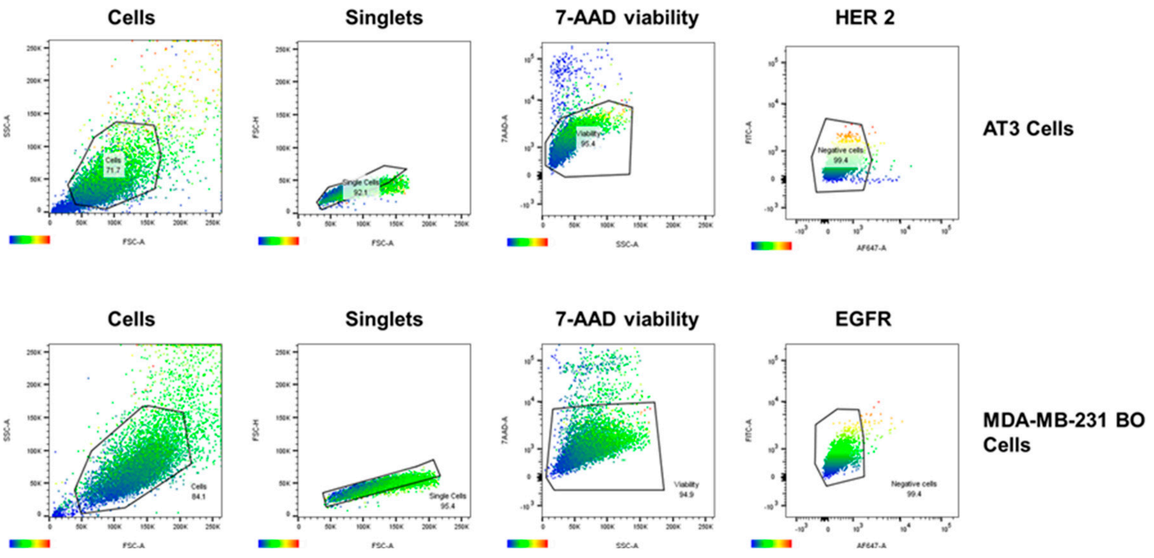

Figure S5. Gating used for flow cytometry analysis of HER 2 and EGFR expression in AT3 and MDA-MB-231 BO cells.

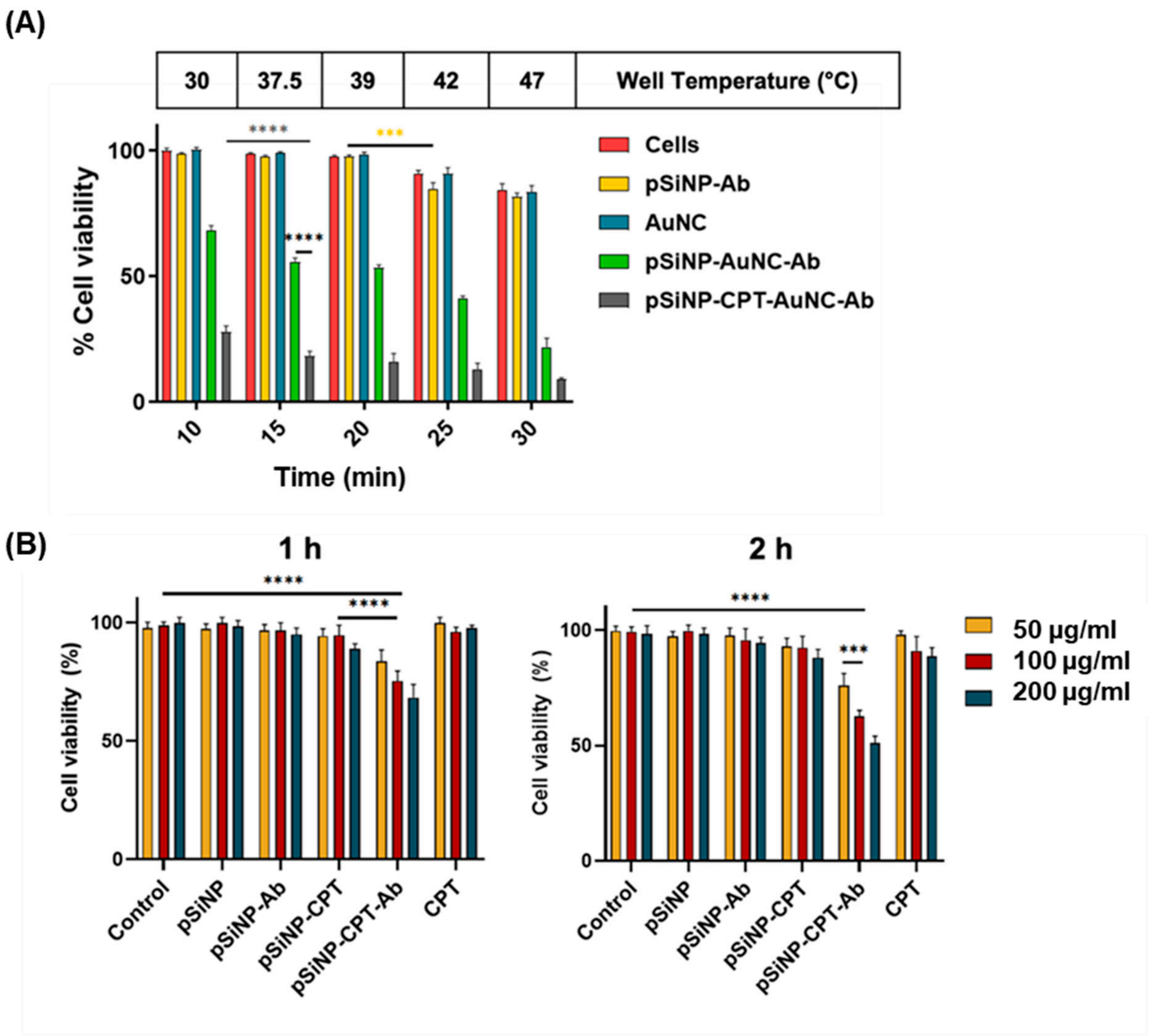

Figure S6. A) Cell viability of AT3 cells upon exposure to RF for the time indicated on x axis after treatment with AuNCs alone and 100 µg/ml pSiNP-Ab with different loads, and corresponding temperature in the well after the indicated RF exposure time. B) Cell viability of AT3 spheroids with different concentrations of pSiNPs with different loads to optimise pSiNP concentration and time of incubation (No pSiNP were added to the control group and for just CPT group, CPT concentration equivalent to amount loaded in respective pSiNP concentration was added to the cells). 'Ab' here refers to anti-HER2 antibody. Data shown as mean ±S.D. (n=3, *** p≤0.001, ****p≤0.0001).

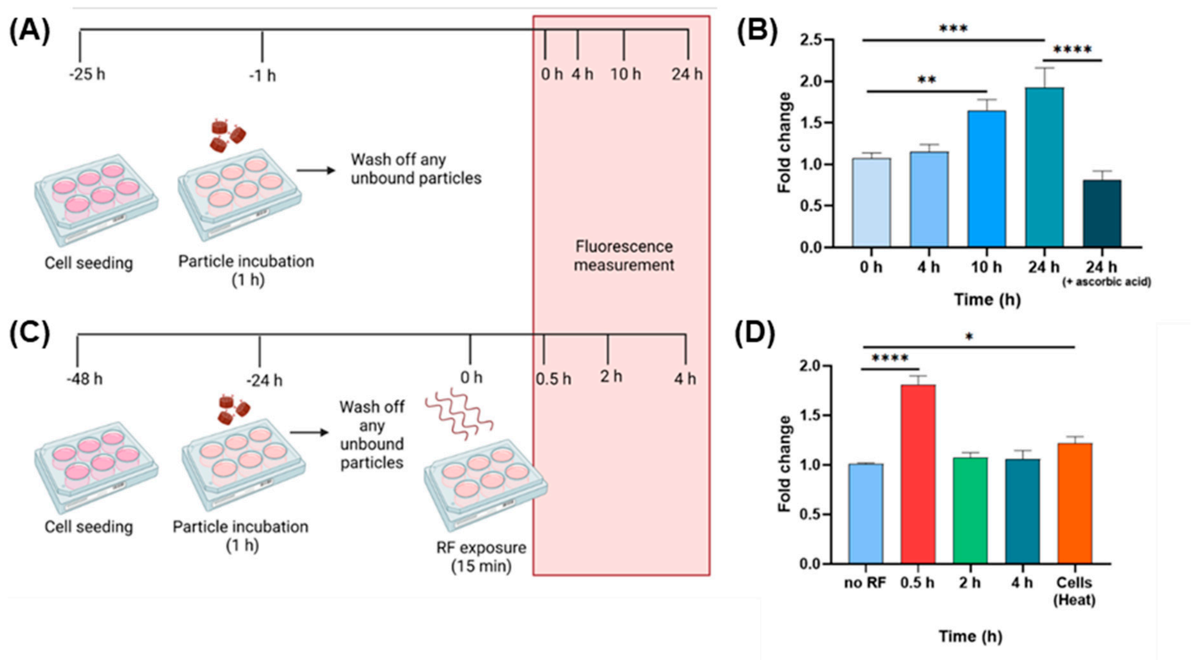

Figure S7. A) Schematic for the experimental flow for determination of ROS generation in AT3 cells by pSiNP-CPT-Ab; B) ROS generation in AT3 cells after 1 h incubation with 100 µg/ml pSiNP-CPT-Ab; Fluorescence signals for ROS were recorded after 0 h, 4 h, 10 h and 24 h; C) Schematic for the experimental flow for determination of ROS generation in AT3 cells by pSiNP-AuNC-Ab nanoparticles D) ROS generation in cells treated with 100 µg/ml pSiNP-AuNC-Ab after 15 min RF exposure (fluorescence recorded at 0.5 h, 2 h and 4h after cells were treated with RF) The term 'Ab' here refers to anti-HER2 antibody. (n=3, ** p≤0.01, *** p≤0.001, **** p≤0.0001).
